# Supplementary material for: Synaptic high-frequency jumping synchronises vision to high-speed behaviour
Source: Nat Commun. 2026 May 5;17:3863. doi: 10.1038/s41467-026-72509-2 (PMC13144400; doi:10.1038/s41467-026-72509-2)
Supplement: Supplementary file 2 — Description of Additional Supplementary Files [file 41467_2026_72509_MOESM2_ESM.pdf]

Description of Additional Supplementary Files

File name: Supplementary Movie 1
Description: LMC response dynamics reveal synaptic high-frequency jumping.
Intracellular voltage recordings from *Musca* first visual interneurons, the large monopolar cells (LMCs) during controlled light stimulation with sharp microelectrodes. Morphodynamic photoreceptor inputs, evoked by high-contrast bursty stimuli mimicking saccadic light fluctuations, are transformed into virtually noise-free LMC responses with minimal delay. These responses exhibit an effective signalling bandwidth of ~920 Hz (signal-to-noise ratio >1), showing how synaptic high-frequency jumping enables rapid, high-fidelity vision at ~0.5 ms resolution.

File name: Supplementary Movie 2
Description: Photomechanical photoreceptor microsaccades drive hyperacute vision.
High-speed infrared imaging of photoreceptor microsaccades, combined with intracellular recordings to high-resolution moving gratings, reveals how *Musca* photoreceptors resolve fine image detail. Each compound eye (~3,500 ommatidia; cf. 800 in *Drosophila*) is wired in neural superposition. Light changes trigger photomechanical microsaccades - tiny receptor contractions that shift receptive fields and actively sample the scene. These movements, smaller and faster in *Musca*, follow evolutionary scaling laws. Grating tests show that Musca photoreceptors resolve features down to 0.9°, far beyond their 2.9° interommatidial angle, with directional tuning. Thus, insect eyes enhance acuity and speed by encoding space dynamically through time.

File name: Supplementary Movie 3
Description: Ultrafast behavioural responses to looming stimuli.
This video shows how Musca react to looming stimuli. A custom arena with high-speed projection and video recording revealed that tethered flies often initiated movements within ~20 ms, with the fastest at 16.8 ms - far shorter than predicted by models requiring ≥4 synapses before motor activation. Connectomic simulations, using conventional conduction and synaptic transmission values for the shortest pathway (photoreceptors → LMCs → optic lobes → giant fibre → TTM “jump muscle”), predict transmission completing in ~22 ms. Most responses, however, occurred later (40-100 ms), implying additional integration and feedback. These results highlight synaptic high-frequency jumping, predictive coding, and rapid neural synchronisation.

File name: Supplementary Movie 4
Description: Simulated conduction of the light-induced antenna reflex pathway in *Musca*.
This video shows a connectome-based simulation of signal transmission from photoreceptors to antennal motor neurons, using conventional conduction and synaptic values from the literature. Light is first transduced by R1-R6 photoreceptors (~10 ms), followed by transmission across six synapses (~6 ms; cf. **Supplementary Fig. 43**). The signal then propagates along >2,640 μm of axon, with conduction speeds of 0.2-0.5 m/s, adding >5.3-13.2 ms. These stages yield a total predicted reflex delay of ~21-29 ms. By contrast, experiments show the fastest light-pulse-triggered antennal movements within 13 ms.
